# Synchronous Endometrioid Adenocarcinoma of the Ovary and Endometrium in a 37-Year-Old Nulligravid Woman: A Case Report

**DOI:** 10.7759/cureus.110669

**Published:** 2026-06-11

**Authors:** Kavya Manivasagham, Aparna Nair, Indhu S, Thejashree KT

**Affiliations:** 1 Obstetrics and Gynaecology, Vydehi Institute of Medical Sciences and Research Centre, Bangalore, IND

**Keywords:** endometrioid adenocarcinoma, endometrium, nulligravid woman, ovary, synchronous malignancy

## Abstract

Synchronous primary malignancies of the endometrium and ovary are uncommon and require careful differentiation from metastatic disease due to differences in staging, management, and prognosis. We report the case of a 37-year-old nulligravid woman presenting with lower abdominal pain and long-standing menstrual irregularity. Imaging revealed a large left adnexal mass with endometrial thickening. Exploratory laparotomy with intraoperative frozen section suggested malignancy, following which comprehensive surgical staging was performed. Final histopathology confirmed synchronous primary endometrioid adenocarcinoma of the ovary (International Federation of Gynecology and Obstetrics (FIGO) IA, grade I) and endometrium (FIGO II, grade II) without nodal or peritoneal involvement. Immunohistochemistry supported independent primary tumors. The patient had an uneventful recovery. This case emphasizes the importance of thorough surgical staging and meticulous pathological evaluation in establishing synchronous primary disease and guiding appropriate management.

## Introduction

Synchronous primary malignancies of the endometrium and ovary constitute a rare yet clinically important entity in gynaecologic oncology, accounting for a small proportion of genital tract cancers [1]. The coexistence of these tumors presents a diagnostic challenge, as they must be carefully distinguished from metastatic disease involving either organ [2]. This distinction is crucial because staging, therapeutic strategies, and prognostic outcomes differ significantly between synchronous primary tumors and metastatic disease [3]. Accurate identification, therefore, has direct implications for patient management and survival [4].

Epidemiologically, synchronous endometrial and ovarian cancers are more frequently observed in younger, premenopausal women compared to isolated malignancies [5]. These tumors are most commonly of endometrioid histology and are often diagnosed at an early stage, which contributes to their relatively favorable prognosis [6]. Shared etiological factors such as obesity, chronic anovulation, hyperestrogenic states, and metabolic disorders, including diabetes mellitus, have been implicated in the pathogenesis of both malignancies [7]. These overlapping risk factors further support the concept of a common pathogenic pathway in synchronous tumor development [8].

The differentiation between synchronous primary tumors and metastatic disease relies predominantly on comprehensive surgical staging and meticulous histopathological evaluation [9]. Established clinicopathological criteria, including tumor grade, depth of myometrial invasion, laterality of ovarian involvement, and absence of lymphovascular or nodal spread, play a pivotal role in this distinction [10]. The application of updated International Federation of Gynecology and Obstetrics (FIGO) staging guidelines enhances diagnostic accuracy and ensures appropriate classification of disease extent [10, 11]. In addition, immunohistochemical and, more recently, molecular analyses have emerged as valuable adjuncts in challenging cases, aiding in the identification of independent primary tumors [12].

In this context, we report a case of synchronous endometrioid adenocarcinoma of the ovary and endometrium in a 37-year-old nulligravid woman, highlighting the importance of thorough surgical staging, detailed pathological assessment, and the role of ancillary diagnostic techniques in establishing an accurate diagnosis.

## Case presentation

A 37-year-old nulligravid woman presented to the gynecology outpatient department with a complaint of lower abdominal pain for 10 days. The pain was insidious in onset, predominantly right-sided, spasmodic in nature, and progressively increasing in intensity. It was partially relieved by ambulation and not associated with vomiting or altered bowel habits. She also reported irregular menstrual cycles for the past five years, occurring at intervals of two to three months, with reduced menstrual flow and occasional passage of clots. There was no history of intermenstrual or postcoital bleeding. She had not undergone any infertility treatment. Her medical history was significant for newly diagnosed type 2 diabetes mellitus detected during evaluation. There was no significant family history of malignancy.

On general examination, the patient was alert, oriented, and hemodynamically stable. Her body mass index was 42.2 kg/m², indicating morbid obesity. Abdominal examination revealed a soft abdomen with a palpable, mobile cystic mass measuring approximately 15 × 10 cm arising from the left iliac region and extending across the midline. Mild tenderness was noted on deep palpation. Speculum examination was limited due to active bleeding, and a foul-smelling vaginal discharge was noted. Bimanual examination demonstrated fullness of the anterior and bilateral fornices. Rectal examination revealed no abnormality.

Baseline laboratory investigations demonstrated mild anemia with a hemoglobin of 11.5 g/dL. Renal and liver function parameters were within normal limits. Glycemic assessment showed poor control with a glycated hemoglobin (HbA1c) of 9%. Serum cancer antigen 125 (CA-125) was mildly elevated at 56.3 U/mL. Serum alpha-fetoprotein, carcinoembryonic antigen, and beta-human chorionic gonadotropin levels were within normal ranges (Table 1).

**Table 1:** Laboratory Investigations

Parameter	Patient Value	Reference Range
Hemoglobin	11.5 g/dL	12–15 g/dL
Glycated hemoglobin	9%	<5.7% (normal)
Cancer antigen 125	56.3 U/mL	<35 U/mL
Alpha-fetoprotein	3.2 ng/mL	<10 ng/mL
Carcinoembryonic antigen	1.8 ng/mL	<3 ng/mL
Beta-human chorionic gonadotropin	<2 mIU/mL	<5 mIU/mL
Blood urea	28 mg/dL	15–40 mg/dL
Serum creatinine	0.8 mg/dL	0.6–1.2 mg/dL
Serum sodium	138 mEq/L	135–145 mEq/L
Serum potassium	4.2 mEq/L	3.5–5.0 mEq/L
Total bilirubin	0.9 mg/dL	0.3–1.2 mg/dL
Aspartate aminotransferase	24 U/L	10–40 U/L
Alanine aminotransferase	26 U/L	7–56 U/L
Alkaline phosphatase	98 U/L	44–147 U/L
Serum albumin	4.1 g/dL	3.5–5.0 g/dL

Pelvic ultrasonography revealed a heterogeneous mass in the left adnexa measuring 10.7 × 7.1 × 9.1 cm, with non-visualisation of the left ovary. A smaller heterogeneous lesion was noted adjacent to the right ovary. The uterus measured 10.7 × 4.7 × 6.9 cm, with an endometrial thickness of 15.7 mm (Figure 1).

**Figure 1:**
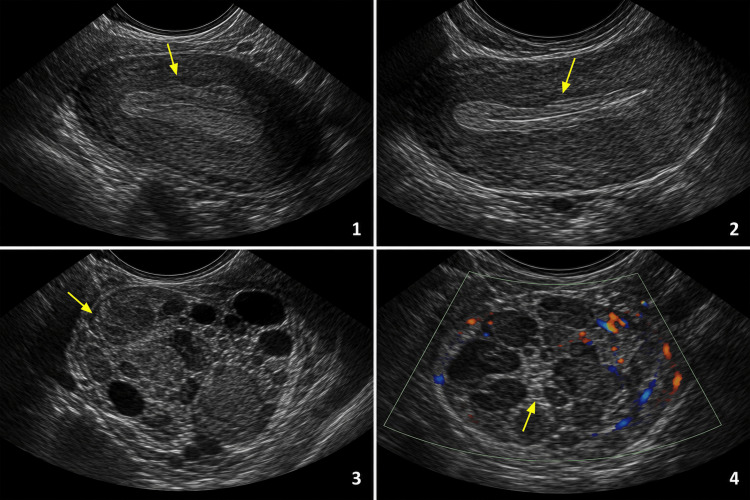
Pelvic ultrasonography images (A) Enlarged uterus with preserved myometrial echotexture (yellow arrow); (B) Diffuse endometrial thickening (yellow arrow); (C) Heterogeneous left adnexal mass with mixed solid and cystic components (yellow arrow); (D) Color Doppler image demonstrating a heterogeneous lesion adjacent to the right ovary with minimal internal vascularity (yellow arrow).

Magnetic resonance imaging demonstrated a large solid-cystic abdominopelvic mass measuring 18 × 10 × 16 cm arising from the left adnexa, consistent with an ovarian neoplasm, along with moderate hydrometrocolpos (Figures 2-7).

**Figure 2:**
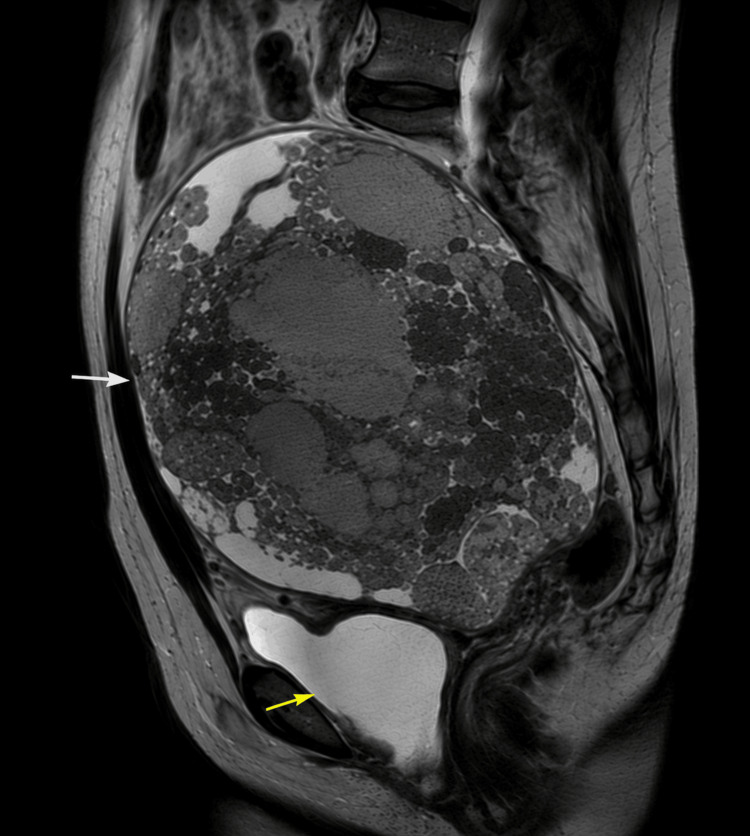
Sagittal T2-weighted magnetic resonance image Large heterogeneous solid-cystic abdominopelvic mass arising from the left adnexa (white arrow) with associated hydrometrocolpos (yellow arrow).

**Figure 3:**
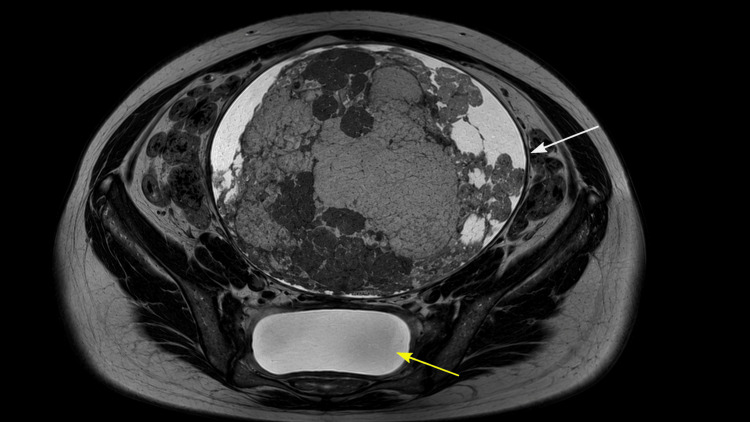
Axial T2-weighted magnetic resonance image Heterogeneous left adnexal solid-cystic mass (white arrow) with associated hydrometrocolpos (yellow arrow).

**Figure 4:**
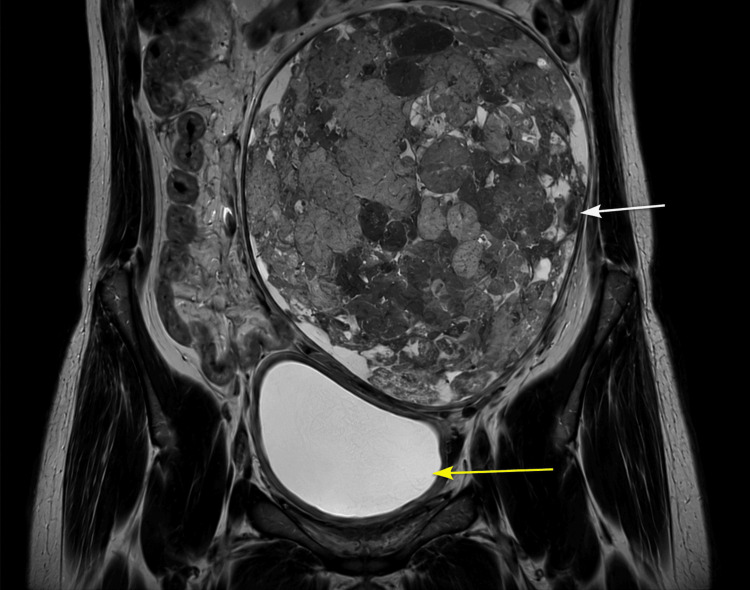
Coronal T2-weighted magnetic resonance image Cranio-caudal extent of the large left adnexal mass (white arrow) with associated hydrometrocolpos (yellow arrow).

**Figure 5:**
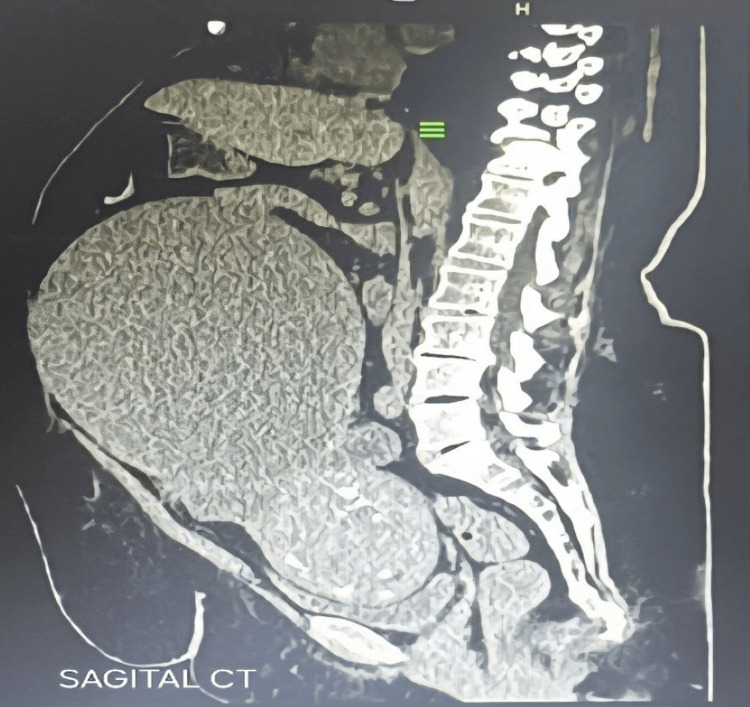
Sagittal CT showing a large abdominopelvic cystic lesion.

**Figure 6:**
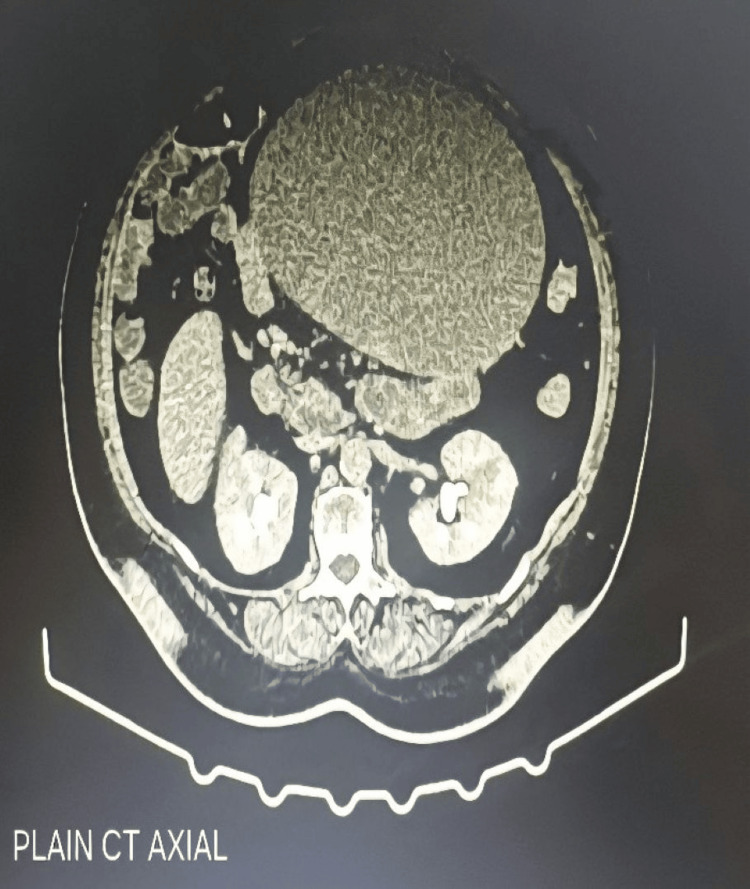
Axial plain CT showing well-defined cystic mass in the left adnexa.

**Figure 7:**
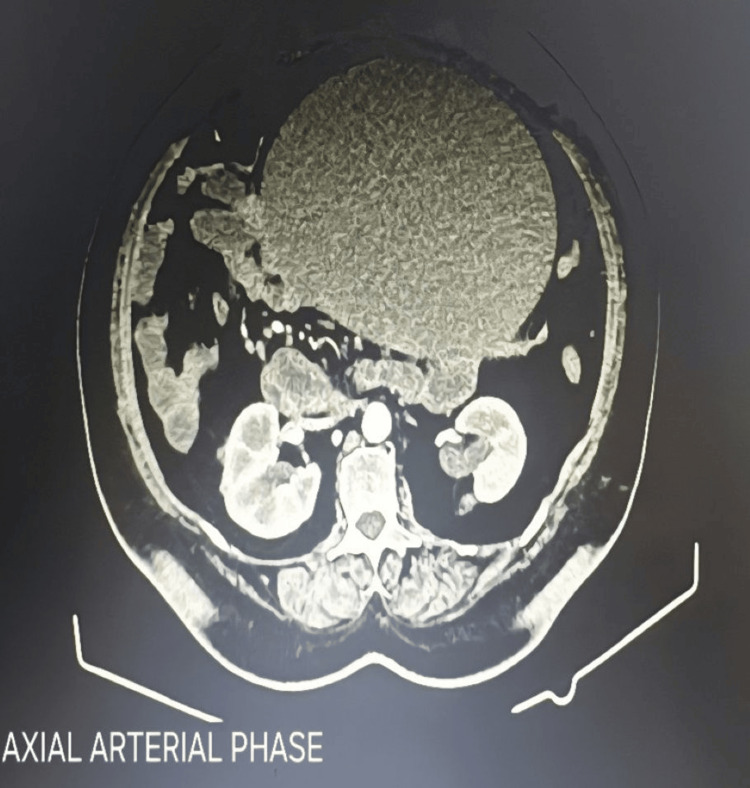
Axial arterial phase showing mass effect with displacement of bowel loops.

In view of the imaging findings and clinical suspicion of malignancy, an exploratory laparotomy was undertaken. Intraoperatively, a large left ovarian cyst measuring approximately 12 × 15 cm with multiple septations and solid areas was identified, completely replacing the ovary. Minimal hemorrhagic ascitic fluid was present and sent for cytological examination. The cyst was carefully exteriorized, and a left ovarian cystectomy was performed. Frozen section analysis revealed malignant epithelial cells suggestive of carcinoma (Figure 8).

**Figure 8:**
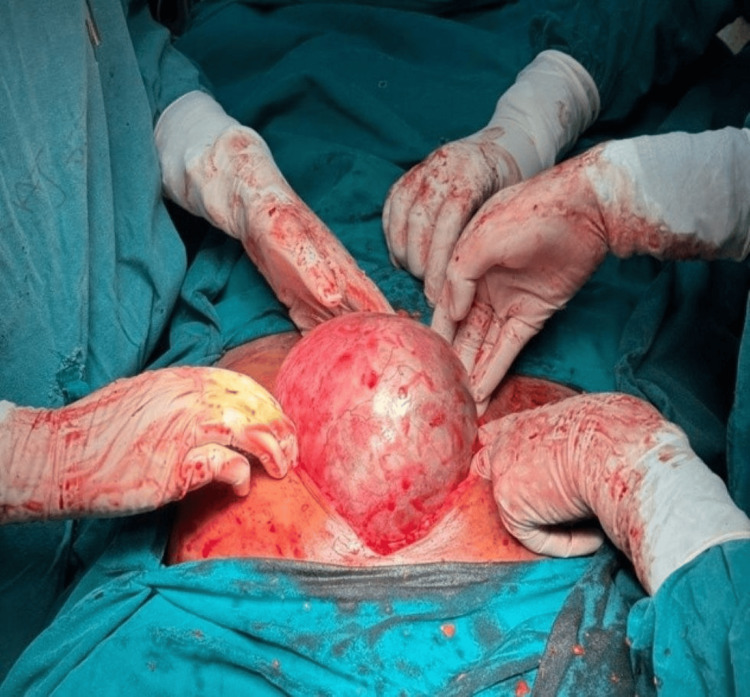
A 12x15 cm left ovarian cyst involving the ovary completely.

Based on frozen section findings, comprehensive surgical staging was completed, including total abdominal hysterectomy with bilateral salpingo-oophorectomy, pelvic lymph node sampling, partial omentectomy, appendicectomy, and peritoneal biopsies from all quadrants. The estimated blood loss was approximately 1500 mL, and the patient received intraoperative transfusion support. The postoperative period was uneventful, and recovery was satisfactory.

Final histopathological evaluation of the left ovarian mass revealed endometrioid adenocarcinoma, grade I, confined to the ovary, corresponding to FIGO stage IA according to the revised FIGO staging system [10]. Examination of the uterus demonstrated grade II endometrioid adenocarcinoma with less than 50% myometrial invasion and involvement of the cervical stroma, consistent with FIGO stage II disease (Figure 9).

**Figure 9:**
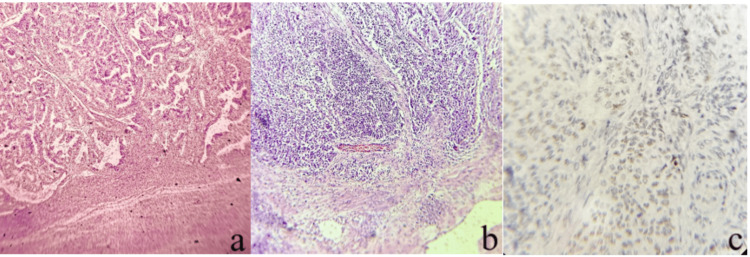
a. Tumor invading myometrium; b. Tumor with adjacent ovarian stroma; c. Immunohistochemistry showing normal nuclear staining.

Peritoneal fluid cytology was negative for malignant cells. Pelvic lymph nodes showed reactive changes without evidence of metastatic involvement. The omentum, appendix, and peritoneal biopsies were free of tumour. Immunohistochemical analysis demonstrated wild-type p53 expression, supporting the diagnosis of synchronous primary tumours rather than metastatic disease (Figures 10-15).

**Figure 10:**
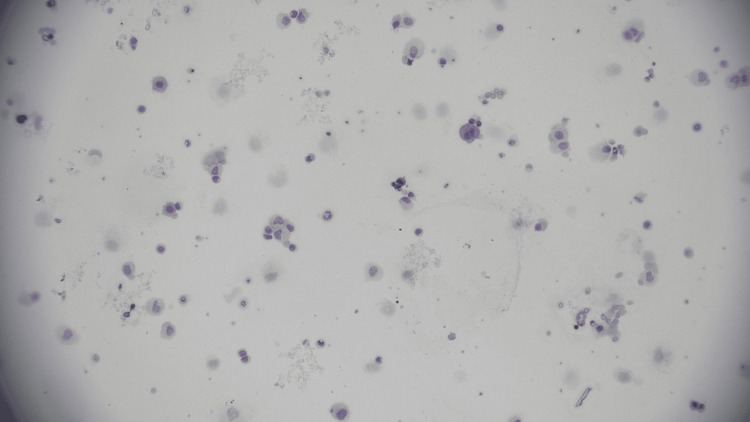
Peritoneal fluid cytology showing reactive mesothelial cells without any evidence of malignancy (H&E stain, 10x magnification).

**Figure 11:**
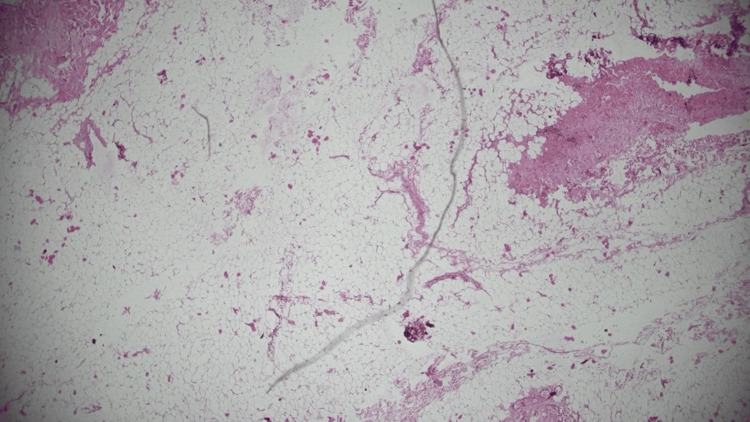
Section from the peritoneum demonstrating predominantly mature adipose tissue negative for malignant cells (H&E stain, 4X magnification).

**Figure 12:**
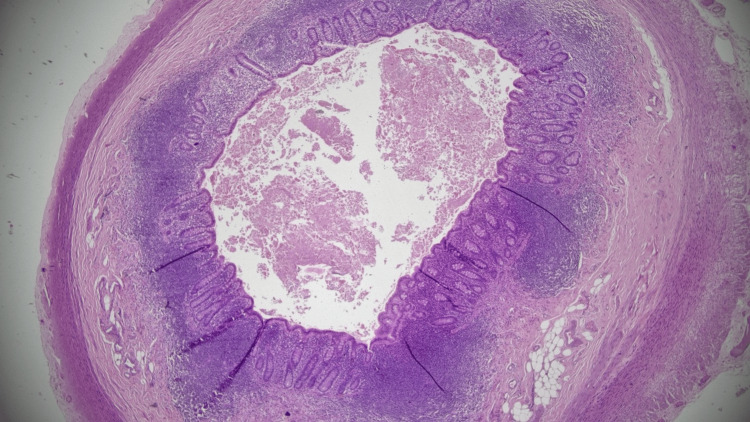
Cross-section of appendix showing no dysplasia or malignant infiltration (H&E stain, 4X magnification).

**Figure 13:**
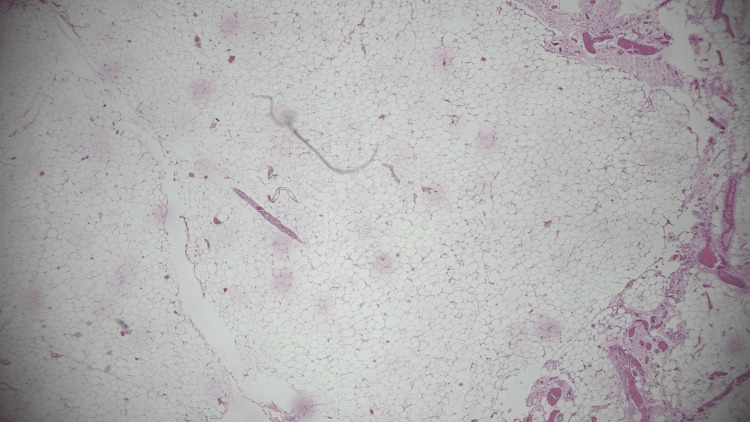
Section from the omentum demonstrating predominantly mature adipose tissue negative for malignant cells (H&E stain, 4X magnification).

**Figure 14:**
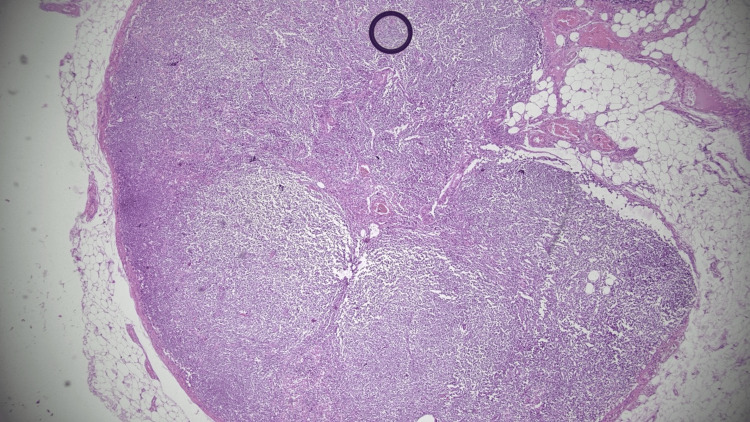
Section from the pelvic lymph node showing preserved nodal architecture with reactive lymphoid hyperplasia and no evidence of metastatic or primary malignancy (H&E stain, 4X magnification).

**Figure 15:**
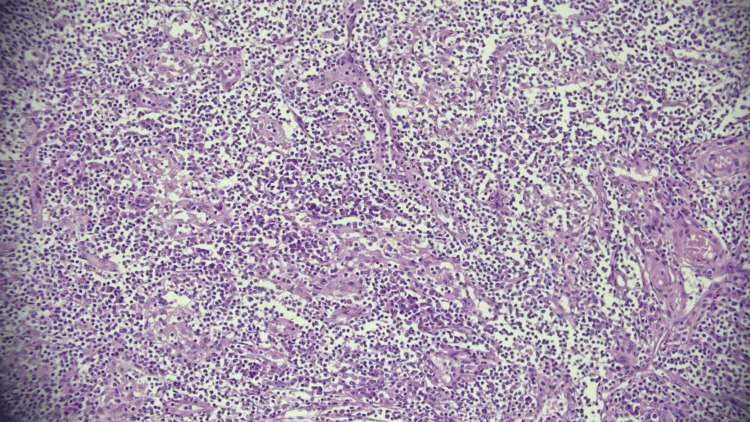
Section from the pelvic lymph node showing reactive lymphoid hyperplasia and no evidence of metastatic or primary malignancy (H&E stain, 20X magnification).

The patient was counselled regarding the diagnosis and referred to the oncology department for further adjuvant management and follow-up planning.

## Discussion

Synchronous primary malignancies of the endometrium and ovary represent a well-recognized but relatively uncommon entity in gynecologic oncology and are generally associated with more favorable outcomes compared to metastatic disease involving both organs [13]. The distinction between synchronous primary tumors and metastatic spread is of paramount importance, as it directly influences staging, therapeutic decision-making, and overall prognosis. Patients with synchronous tumors typically present with early-stage disease and demonstrate improved survival outcomes, underscoring the need for accurate diagnosis and appropriate classification [13].

These malignancies most commonly exhibit endometrioid histology and are frequently encountered in younger or premenopausal women. The present case aligns with existing literature, as the patient was relatively young and demonstrated risk factors such as obesity and metabolic dysfunction, including diabetes mellitus [14]. Shared etiological factors such as hyperestrogenism, chronic anovulation, and metabolic syndrome have been implicated in the pathogenesis of both endometrial and ovarian endometrioid carcinomas, suggesting a possible common hormonal and molecular pathway [14]. This overlapping risk profile further supports the concept of synchronous tumor development rather than metastatic disease.

The differentiation between synchronous primary tumors and metastatic disease relies heavily on well-established clinicopathological criteria. Features favoring synchronous primaries include unilateral ovarian involvement, low-grade histology, superficial myometrial invasion, absence of lymphovascular space invasion, and lack of nodal or distant metastasis [10]. In the present case, the ovarian tumor was confined to a single ovary and exhibited low-grade morphology, while the endometrial carcinoma demonstrated limited myometrial invasion without lymph node involvement or distant spread [10]. These findings strongly support the diagnosis of independent primary tumors. Furthermore, the absence of a tumor in the omentum, appendix, peritoneal biopsies, and lymph nodes, along with negative peritoneal cytology, reinforces the non-metastatic nature of the disease.

In addition to conventional histopathological assessment, immunohistochemistry plays an important adjunctive role in challenging cases. The demonstration of wild-type p53 expression in the present case supports the diagnosis of synchronous primary tumors, as aberrant p53 expression is more commonly associated with high-grade or metastatic disease. Emerging molecular studies have further highlighted the utility of genetic profiling in distinguishing synchronous tumors from metastatic lesions; however, such techniques may not always be readily available in routine clinical practice [15].

A recent case series by Hoorshad et al. reported that synchronous endometrial and ovarian cancers are often diagnosed at early stages and are associated with favorable clinical outcomes when comprehensive surgical staging and appropriate management are undertaken [13]. The findings in the present case are consistent with these observations, as the patient demonstrated early-stage disease with no evidence of metastatic involvement. This case, therefore, emphasizes the critical role of thorough surgical staging, meticulous pathological evaluation, and appropriate use of adjunctive diagnostic tools in establishing an accurate diagnosis and guiding optimal management.

## Conclusions

This case underscores the importance of maintaining a high index of suspicion for synchronous primary endometrial and ovarian malignancies, particularly in younger women presenting with adnexal masses and menstrual irregularities. Distinguishing synchronous primaries from metastatic disease is critical, as it has direct implications for staging, therapeutic decision-making, and prognostic assessment. A systematic approach integrating comprehensive surgical staging, detailed histopathological evaluation, and adjunctive immunohistochemical analysis is essential for accurate diagnosis. Early recognition and appropriate classification of these tumors enable tailored management strategies and are associated with favorable clinical outcomes.
